# Supplementary material for: Preparation of Graphene/ITO Nanorod Metamaterial/U-Bent-Annealing Fiber Sensor and DNA Biomolecule Detection
Source: Nanomaterials (Basel). 2019 Aug 12;9(8):1154. doi: 10.3390/nano9081154 (PMC6723577; doi:10.3390/nano9081154)
Supplement: Supplementary file 1 [file nanomaterials-09-01154-s001.pdf]

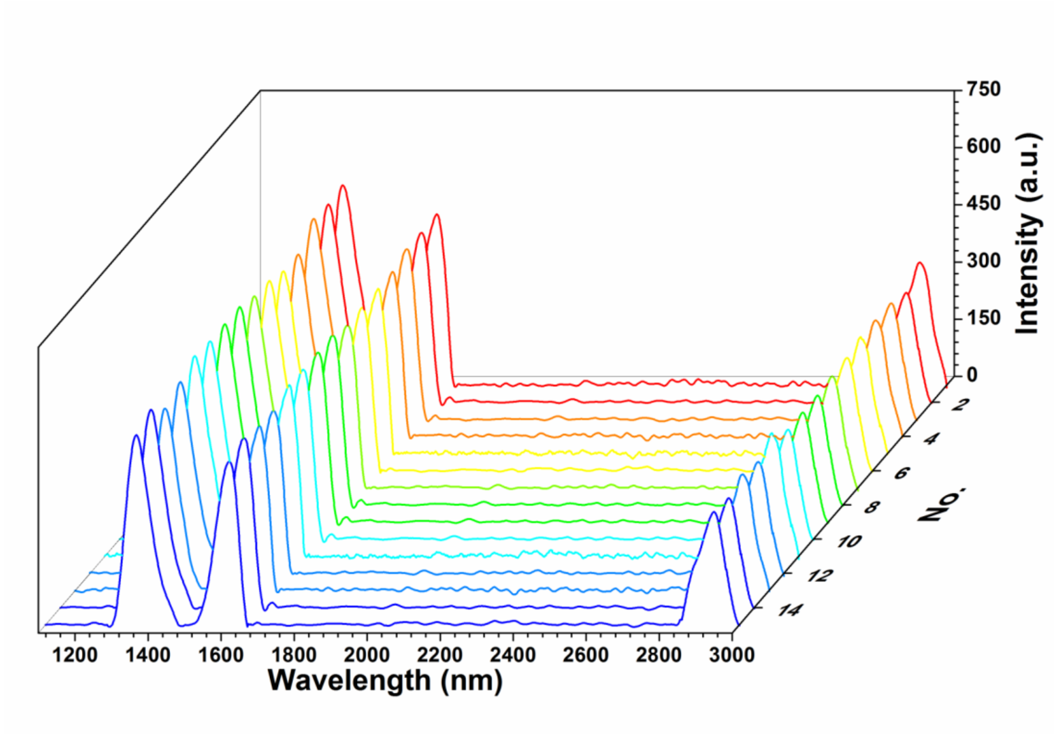

Figure S1. The 15 groups of Raman spectra collected from different sensors.

Graphene is a very common two-dimensional material. In order to ensure the accuracy of the experimental results, we have explored the reproducibility of chemical methods. Figure S1 shows 15 groups of Raman spectra of graphene. In the figure, the peaks of 1380, 1580 and 2910 cm^{-1} peaks indicate that we have prepared graphene, the height of each peaks only have small float. This uniform phenomenon indicates that the chemical method for preparing graphene has good reproducibility.
